# Supplementary material for: Lateral hypothalamus CRFR1 regulation of chronic binge drinking: divergence along anterior-posterior axis
Source: bioRxiv. 2025 Sep 16:2025.09.16.676507. Preprint. [Version 1] doi: 10.1101/2025.09.16.676507 (PMC12458246; doi:10.1101/2025.09.16.676507)
Supplement: 1 [file NIHPP2025.09.16.676507v1-supplement-1.pdf]

bioRxiv preprint doi: https://doi.org/10.1101/2025.09.16.676507; this version posted September 16, 2025. The copyright holder for this preprint (which was not certified by peer review) is the author/funder, who has granted bioRxiv a license to display the preprint in perpetuity. It is made available under aCC-BY 4.0 International license.

Supplemental Figure 1

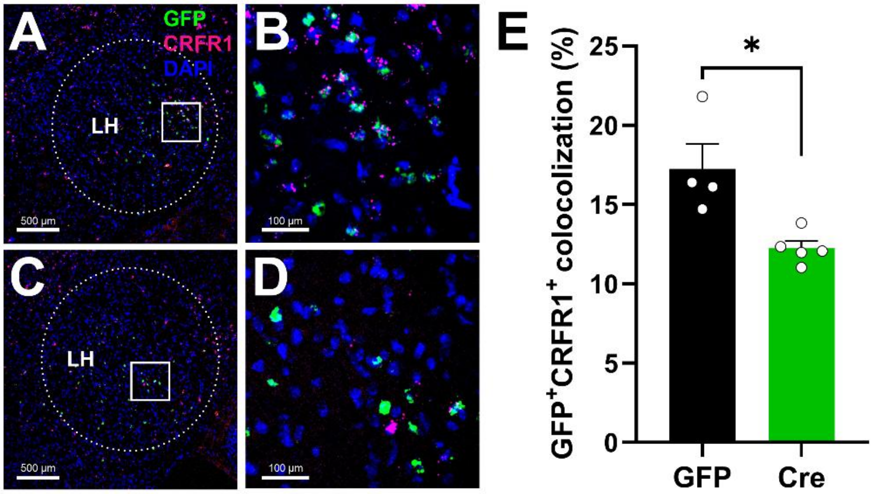

Efficacy of viral knockdown was assessed using FISH labeling *GFP* and *Crhr1* mRNA in tissue sections containing the LH of **(A)** GFP- and **(C)** Cre-virus expressing mice. **(B**, **D)** 20x magnification of the LH (area in white square). **(E)** Quantification of *GFP*$^+$*Crhr1*$^+$ colocalized cells revealed a ~29% reduction in colocalized cells of Cre mice relative to GFP mice (n = 16 slices from 9 mice). Data are represented as mean +/- SEM. *t-test, different from GFP, p = 0.04.
